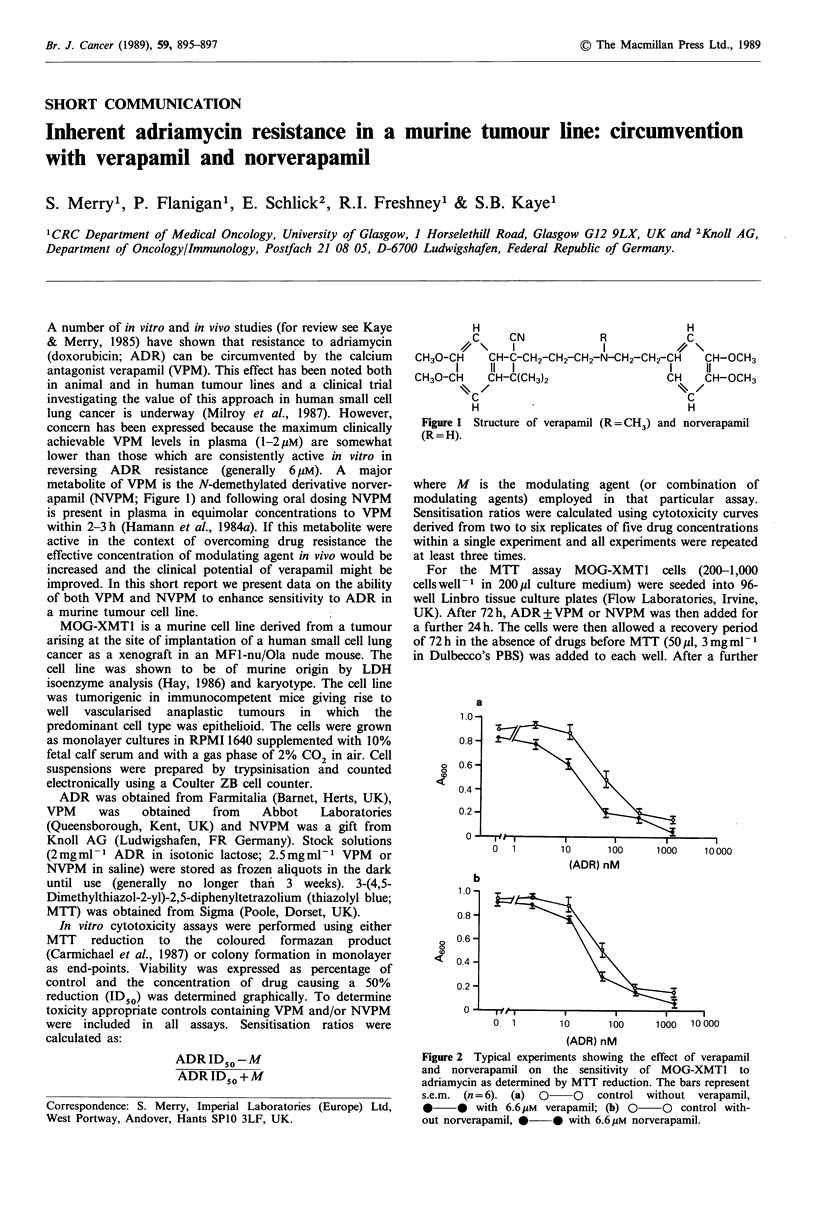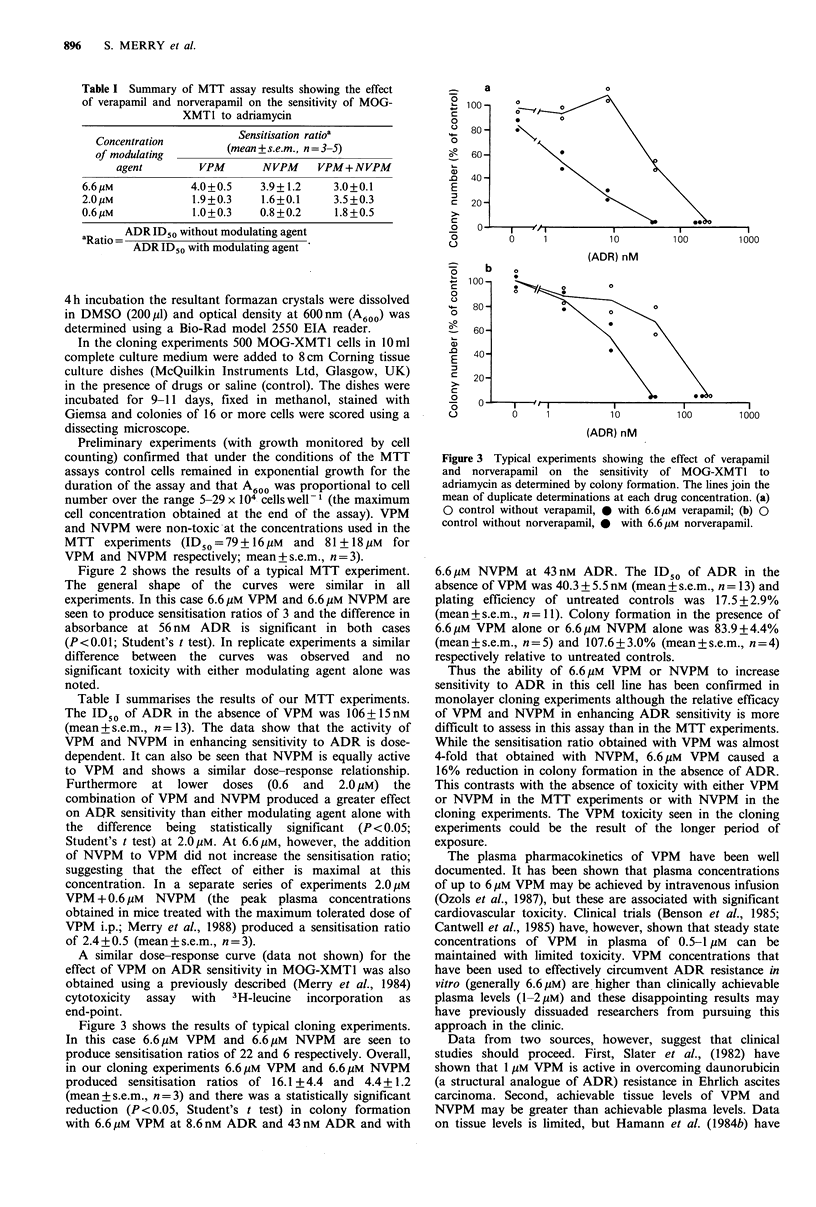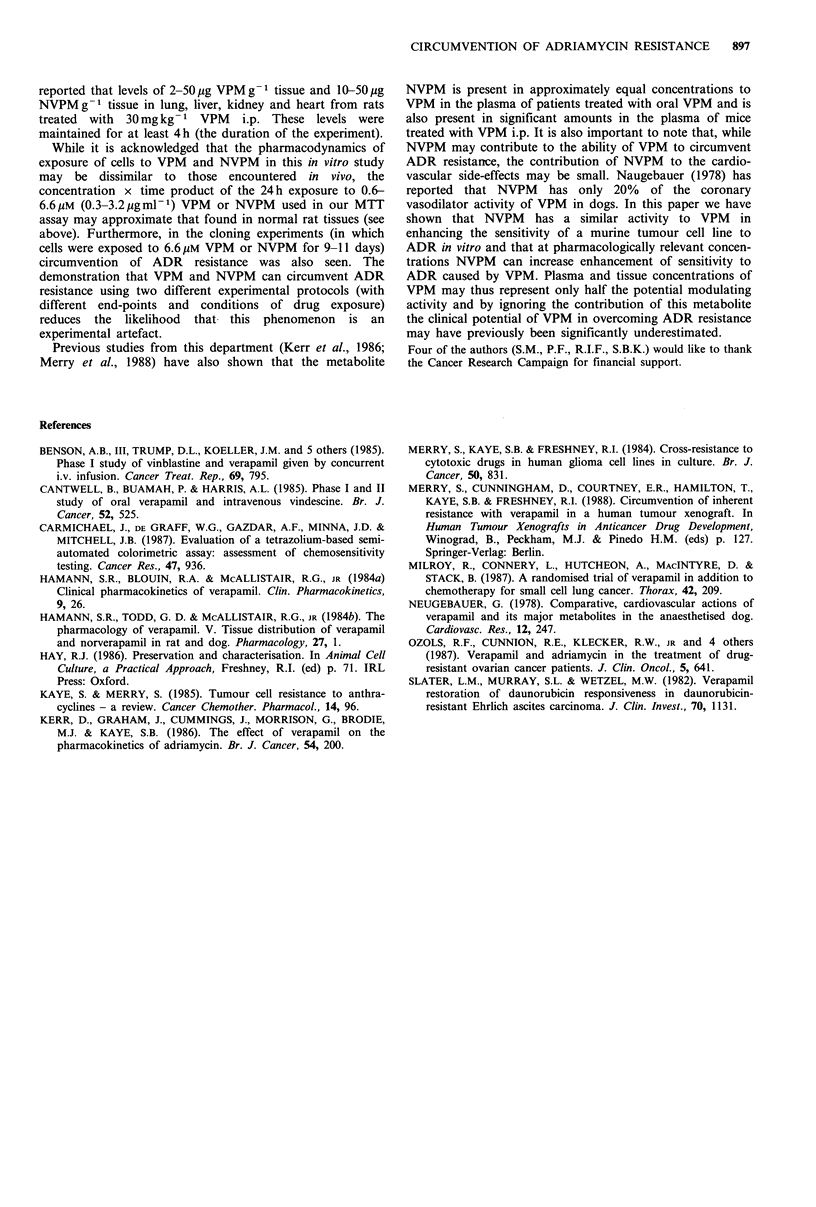# Inherent adriamycin resistance in a murine tumour line: circumvention with verapamil and norverapamil.

**DOI:** 10.1038/bjc.1989.189

**Published:** 1989-06

**Authors:** S. Merry, P. Flanigan, E. Schlick, R. I. Freshney, S. B. Kaye

**Affiliations:** CRC Department of Medical Oncology, University of Glasgow, UK.


Br. J. Cancer (1989), 59, 895–897 © The Macmillan Press Ltd., 1989

SHORT COMMUNICATION

### Inherent adriamycin resistance in a murine tumour line: circumvention with verapamil and norverapamil

S. Merry[1], P. Flanigan[1], E. Schlick[2], R.I. Freshney[1] & S.B. Kaye[1]

[1]*CRC Department of Medical Oncology, University of Glasgow, 1 Horselethill Road, Glasgow G12 9LX, UK and* [2]*Knoll AG, Department of Oncology/Immunology, Postfach 21 08 05, D-6700 Ludwigshafen, Federal Republic of Germany.*

A number of *in vitro* and *in vivo* studies (for review see Kaye & Merry, 1985) have shown that resistance to adriamycin (doxorubicin; ADR) can be circumvented by the calcium antagonist verapamil (VPM). This effect has been noted both in animal and in human tumour lines and a clinical trial investigating the value of this approach in human small cell lung cancer is underway (Milroy *et al.*, 1987). However, concern has been expressed because the maximum clinically achievable VPM levels in plasma (1–2 $\mu$M) are somewhat lower than those which are consistently active *in vitro* in reversing ADR resistance (generally 6 $\mu$M). A major metabolite of VPM is the *N*-demethylated derivative norverapamil (NVPM; Figure 1) and following oral dosing NVPM is present in plasma in equimolar concentrations to VPM within 2–3 h (Hamann *et al.*, 1984*a*). If this metabolite were active in the context of overcoming drug resistance the effective concentration of modulating agent *in vivo* would be increased and the clinical potential of verapamil might be improved. In this short report we present data on the ability of both VPM and NVPM to enhance sensitivity to ADR in a murine tumour cell line.

MOG-XMT1 is a murine cell line derived from a tumour arising at the site of implantation of a human small cell lung cancer as a xenograft in an MF1-nu/Ola nude mouse. The cell line was shown to be of murine origin by LDH isoenzyme analysis (Hay, 1986) and karyotype. The cell line was tumorigenic in immunocompetent mice giving rise to well vascularised anaplastic tumours in which the predominant cell type was epithelioid. The cells were grown as monolayer cultures in RPMI 1640 supplemented with 10% fetal calf serum and with a gas phase of 2% $CO_2$ in air. Cell suspensions were prepared by trypsinisation and counted electronically using a Coulter ZB cell counter.

ADR was obtained from Farmitalia (Barnet, Herts, UK), VPM was obtained from Abbot Laboratories (Queensborough, Kent, UK) and NVPM was a gift from Knoll AG (Ludwigshafen, FR Germany). Stock solutions (2 mg ml$^{-1}$ ADR in isotonic lactose; 2.5 mg ml$^{-1}$ VPM or NVPM in saline) were stored as frozen aliquots in the dark until use (generally no longer than 3 weeks). 3-(4,5-Dimethylthiazol-2-yl)-2,5-diphenyltetrazolium (thiazolyl blue; MTT) was obtained from Sigma (Poole, Dorset, UK).

*In vitro* cytotoxicity assays were performed using either MTT reduction to the coloured formazan product (Carmichael *et al.*, 1987) or colony formation in monolayer as end-points. Viability was expressed as percentage of control and the concentration of drug causing a 50% reduction ($ID_{50}$) was determined graphically. To determine toxicity appropriate controls containing VPM and/or NVPM were included in all assays. Sensitisation ratios were calculated as:

$$\frac{\mathrm{ADR\,ID_{50}} - M}{\mathrm{ADR\,ID_{50}} + M}$$

Correspondence: S. Merry, Imperial Laboratories (Europe) Ltd, West Portway, Andover, Hants SP10 3LF, UK.

**Figure 1** Structure of verapamil (R = $CH_3$) and norverapamil (R = H).

where *M* is the modulating agent (or combination of modulating agents) employed in that particular assay. Sensitisation ratios were calculated using cytotoxicity curves derived from two to six replicates of five drug concentrations within a single experiment and all experiments were repeated at least three times.

For the MTT assay MOG-XMT1 cells (200–1,000 cells well$^{-1}$ in 200 $\mu$l culture medium) were seeded into 96-well Linbro tissue culture plates (Flow Laboratories, Irvine, UK). After 72 h, ADR ± VPM or NVPM was then added for a further 24 h. The cells were then allowed a recovery period of 72 h in the absence of drugs before MTT (50 $\mu$l, 3 mg ml$^{-1}$ in Dulbecco's PBS) was added to each well. After a further

**Figure 2** Typical experiments showing the effect of verapamil and norverapamil on the sensitivity of MOG-XMT1 to adriamycin as determined by MTT reduction. The bars represent s.e.m. ($n$ = 6). (**a**) ○——○ control without verapamil, ●——● with 6.6 $\mu$M verapamil; (**b**) ○——○ control without norverapamil, ●——● with 6.6 $\mu$M norverapamil.

**Table I** Summary of MTT assay results showing the effect of verapamil and norverapamil on the sensitivity of MOG-XMT1 to adriamycin

| *Concentration of modulating agent* | *Sensitisation ratio*[a] *(mean ± s.e.m., n = 3–5)* | | |
|---|---|---|---|
| | *VPM* | *NVPM* | *VPM + NVPM* |
| 6.6 μM | 4.0 ± 0.5 | 3.9 ± 1.2 | 3.0 ± 0.1 |
| 2.0 μM | 1.9 ± 0.3 | 1.6 ± 0.1 | 3.5 ± 0.3 |
| 0.6 μM | 1.0 ± 0.3 | 0.8 ± 0.2 | 1.8 ± 0.5 |

[a]Ratio $= \frac{\text{ADR ID}_{50}\text{ without modulating agent}}{\text{ADR ID}_{50}\text{ with modulating agent}}$.

4 h incubation the resultant formazan crystals were dissolved in DMSO (200 μl) and optical density at 600 nm ($A_{600}$) was determined using a Bio-Rad model 2550 EIA reader.

In the cloning experiments 500 MOG-XMT1 cells in 10 ml complete culture medium were added to 8 cm Corning tissue culture dishes (McQuilkin Instruments Ltd, Glasgow, UK) in the presence of drugs or saline (control). The dishes were incubated for 9–11 days, fixed in methanol, stained with Giemsa and colonies of 16 or more cells were scored using a dissecting microscope.

Preliminary experiments (with growth monitored by cell counting) confirmed that under the conditions of the MTT assays control cells remained in exponential growth for the duration of the assay and that $A_{600}$ was proportional to cell number over the range 5–29 × $10^4$ cells well$^{-1}$ (the maximum cell concentration obtained at the end of the assay). VPM and NVPM were non-toxic at the concentrations used in the MTT experiments ($ID_{50}$ = 79 ± 16 μM and 81 ± 18 μM for VPM and NVPM respectively; mean ± s.e.m., $n = 3$).

Figure 2 shows the results of a typical MTT experiment. The general shape of the curves were similar in all experiments. In this case 6.6 μM VPM and 6.6 μM NVPM are seen to produce sensitisation ratios of 3 and the difference in absorbance at 56 nM ADR is significant in both cases ($P < 0.01$; Student's $t$ test). In replicate experiments a similar difference between the curves was observed and no significant toxicity with either modulating agent alone was noted.

Table I summarises the results of our MTT experiments. The $ID_{50}$ of ADR in the absence of VPM was 106 ± 15 nM (mean ± s.e.m., $n = 13$). The data show that the activity of VPM and NVPM in enhancing sensitivity to ADR is dose-dependent. It can also be seen that NVPM is equally active to VPM and shows a similar dose–response relationship. Furthermore at lower doses (0.6 and 2.0 μM) the combination of VPM and NVPM produced a greater effect on ADR sensitivity than either modulating agent alone with the difference being statistically significant ($P < 0.05$; Student's $t$ test) at 2.0 μM. At 6.6 μM, however, the addition of NVPM to VPM did not increase the sensitisation ratio; suggesting that the effect of either is maximal at this concentration. In a separate series of experiments 2.0 μM VPM + 0.6 μM NVPM (the peak plasma concentrations obtained in mice treated with the maximum tolerated dose of VPM i.p.; Merry *et al.*, 1988) produced a sensitisation ratio of 2.4 ± 0.5 (mean ± s.e.m., $n = 3$).

A similar dose–response curve (data not shown) for the effect of VPM on ADR sensitivity in MOG-XMT1 was also obtained using a previously described (Merry *et al.*, 1984) cytotoxicity assay with $^3$H-leucine incorporation as end-point.

Figure 3 shows the results of typical cloning experiments. In this case 6.6 μM VPM and 6.6 μM NVPM are seen to produce sensitisation ratios of 22 and 6 respectively. Overall, in our cloning experiments 6.6 μM VPM and 6.6 μM NVPM produced sensitisation ratios of 16.1 ± 4.4 and 4.4 ± 1.2 (mean ± s.e.m., $n = 3$) and there was a statistically significant reduction ($P < 0.05$, Student's $t$ test) in colony formation with 6.6 μM VPM at 8.6 nM ADR and 43 nM ADR and with

**Figure 3** Typical experiments showing the effect of verapamil and norverapamil on the sensitivity of MOG-XMT1 to adriamycin as determined by colony formation. The lines join the mean of duplicate determinations at each drug concentration. (**a**) ○ control without verapamil, ● with 6.6 μM verapamil; (**b**) ○ control without norverapamil, ● with 6.6 μM norverapamil.

6.6 μM NVPM at 43 nM ADR. The $ID_{50}$ of ADR in the absence of VPM was 40.3 ± 5.5 nM (mean ± s.e.m., $n = 13$) and plating efficiency of untreated controls was 17.5 ± 2.9% (mean ± s.e.m., $n = 11$). Colony formation in the presence of 6.6 μM VPM alone or 6.6 μM NVPM alone was 83.9 ± 4.4% (mean ± s.e.m., $n = 5$) and 107.6 ± 3.0% (mean ± s.e.m., $n = 4$) respectively relative to untreated controls.

Thus the ability of 6.6 μM VPM or NVPM to increase sensitivity to ADR in this cell line has been confirmed in monolayer cloning experiments although the relative efficacy of VPM and NVPM in enhancing ADR sensitivity is more difficult to assess in this assay than in the MTT experiments. While the sensitisation ratio obtained with VPM was almost 4-fold that obtained with NVPM, 6.6 μM VPM caused a 16% reduction in colony formation in the absence of ADR. This contrasts with the absence of toxicity with either VPM or NVPM in the MTT experiments or with NVPM in the cloning experiments. The VPM toxicity seen in the cloning experiments could be the result of the longer period of exposure.

The plasma pharmacokinetics of VPM have been well documented. It has been shown that plasma concentrations of up to 6 μM VPM may be achieved by intravenous infusion (Ozols *et al.*, 1987), but these are associated with significant cardiovascular toxicity. Clinical trials (Benson *et al.*, 1985; Cantwell *et al.*, 1985) have, however, shown that steady state concentrations of VPM in plasma of 0.5–1 μM can be maintained with limited toxicity. VPM concentrations that have been used to effectively circumvent ADR resistance *in vitro* (generally 6.6 μM) are higher than clinically achievable plasma levels (1–2 μM) and these disappointing results may have previously dissuaded researchers from pursuing this approach in the clinic.

Data from two sources, however, suggest that clinical studies should proceed. First, Slater *et al.*, (1982) have shown that 1 μM VPM is active in overcoming daunorubicin (a structural analogue of ADR) resistance in Ehrlich ascites carcinoma. Second, achievable tissue levels of VPM and NVPM may be greater than achievable plasma levels. Data on tissue levels is limited, but Hamann *et al.* (1984*b*) have reported that levels of 2–50 $\mu g$ VPM $g^{-1}$ tissue and 10–50 $\mu g$ NVPM $g^{-1}$ tissue in lung, liver, kidney and heart from rats treated with 30 $mg\,kg^{-1}$ VPM i.p. These levels were maintained for at least 4 h (the duration of the experiment).

While it is acknowledged that the pharmacodynamics of exposure of cells to VPM and NVPM in this *in vitro* study may be dissimilar to those encountered *in vivo*, the concentration × time product of the 24 h exposure to 0.6–6.6 $\mu M$ (0.3–3.2 $\mu g\,ml^{-1}$) VPM or NVPM used in our MTT assay may approximate that found in normal rat tissues (see above). Furthermore, in the cloning experiments (in which cells were exposed to 6.6 $\mu M$ VPM or NVPM for 9–11 days) circumvention of ADR resistance was also seen. The demonstration that VPM and NVPM can circumvent ADR resistance using two different experimental protocols (with different end-points and conditions of drug exposure) reduces the likelihood that this phenomenon is an experimental artefact.

Previous studies from this department (Kerr *et al.*, 1986; Merry *et al.*, 1988) have also shown that the metabolite NVPM is present in approximately equal concentrations to VPM in the plasma of patients treated with oral VPM and is also present in significant amounts in the plasma of mice treated with VPM i.p. It is also important to note that, while NVPM may contribute to the ability of VPM to circumvent ADR resistance, the contribution of NVPM to the cardiovascular side-effects may be small. Naugebauer (1978) has reported that NVPM has only 20% of the coronary vasodilator activity of VPM in dogs. In this paper we have shown that NVPM has a similar activity to VPM in enhancing the sensitivity of a murine tumour cell line to ADR *in vitro* and that at pharmacologically relevant concentrations NVPM can increase enhancement of sensitivity to ADR caused by VPM. Plasma and tissue concentrations of VPM may thus represent only half the potential modulating activity and by ignoring the contribution of this metabolite the clinical potential of VPM in overcoming ADR resistance may have previously been significantly underestimated.

Four of the authors (S.M., P.F., R.I.F., S.B.K.) would like to thank the Cancer Research Campaign for financial support.

### References

BENSON, A.B., III, TRUMP, D.L., KOELLER, J.M. and 5 others (1985). Phase I study of vinblastine and verapamil given by concurrent i.v. infusion. *Cancer Treat. Rep.*, **69**, 795.

CANTWELL, B., BUAMAH, P. & HARRIS, A.L. (1985). Phase I and II study of oral verapamil and intravenous vindescine. *Br. J. Cancer*, **52**, 525.

CARMICHAEL, J., DE GRAFF, W.G., GAZDAR, A.F., MINNA, J.D. & MITCHELL, J.B. (1987). Evaluation of a tetrazolium-based semi-automated colorimetric assay: assessment of chemosensitivity testing. *Cancer Res.*, **47**, 936.

HAMANN, S.R., BLOUIN, R.A. & MCALLISTAIR, R.G., JR (1984*a*) Clinical pharmacokinetics of verapamil. *Clin. Pharmacokinetics*, **9**, 26.

HAMANN, S.R., TODD, G. D. & MCALLISTAIR, R.G., JR (1984*b*). The pharmacology of verapamil. V. Tissue distribution of verapamil and norverapamil in rat and dog. *Pharmacology*, **27**, 1.

HAY, R.J. (1986). Preservation and characterisation. In *Animal Cell Culture, a Practical Approach*, Freshney, R.I. (ed) p. 71. IRL Press: Oxford.

KAYE, S. & MERRY, S. (1985). Tumour cell resistance to anthracyclines – a review. *Cancer Chemother. Pharmacol.*, **14**, 96.

KERR, D., GRAHAM, J., CUMMINGS, J., MORRISON, G., BRODIE, M.J. & KAYE, S.B. (1986). The effect of verapamil on the pharmacokinetics of adriamycin. *Br. J. Cancer*, **54**, 200.

MERRY, S., KAYE, S.B. & FRESHNEY, R.I. (1984). Cross-resistance to cytotoxic drugs in human glioma cell lines in culture. *Br. J. Cancer*, **50**, 831.

MERRY, S., CUNNINGHAM, D., COURTNEY, E.R., HAMILTON, T., KAYE, S.B. & FRESHNEY, R.I. (1988). Circumvention of inherent resistance with verapamil in a human tumour xenograft. In *Human Tumour Xenografts in Anticancer Drug Development*, Winograd, B., Peckham, M.J. & Pinedo H.M. (eds) p. 127. Springer-Verlag: Berlin.

MILROY, R., CONNERY, L., HUTCHEON, A., MACINTYRE, D. & STACK, B. (1987). A randomised trial of verapamil in addition to chemotherapy for small cell lung cancer. *Thorax*, **42**, 209.

NEUGEBAUER, G. (1978). Comparative, cardiovascular actions of verapamil and its major metabolites in the anaesthetised dog. *Cardiovasc. Res.*, **12**, 247.

OZOLS, R.F., CUNNION, R.E., KLECKER, R.W., JR and 4 others (1987). Verapamil and adriamycin in the treatment of drug-resistant ovarian cancer patients. *J. Clin. Oncol.*, **5**, 641.

SLATER, L.M., MURRAY, S.L. & WETZEL, M.W. (1982). Verapamil restoration of daunorubicin responsiveness in daunorubicin-resistant Ehrlich ascites carcinoma. *J. Clin. Invest.*, **70**, 1131.